# How Course Support and Academic Support Impact on Chinese Graduate Students during the COVID-19: The Multiple Mediating Roles of Thesis Writing and Anxiety

**DOI:** 10.3390/ijerph19010265

**Published:** 2021-12-27

**Authors:** Zhengyan Liang, Qing Zeng, Minqiang Zhang, Huijun Luo, Sijuan Huang, Jia Li, Da Yi

**Affiliations:** 1 School of Psychology, South China Normal University, Guangzhou 510631, China; 2020010220@m.scnu.edu.cn (Z.L.); 2020023457@m.scnu.edu.cn (Q.Z.); 2021023799@m.scnu.edu.cn (H.L.); 2021023740@m.scnu.edu.cn (S.H.); 2020023510@m.scnu.edu.cn (J.L.); 2020023499@m.scnu.edu.cn (D.Y.); 2 Key Laboratory of Brain, Cognition and Education Sciences, Ministry of Education, South China Normal University, Guangzhou 510631, China; 3 Center for Studies of Psychological Application, School of Psychology, South China Normal University, Guangzhou 510631, China; 4 Guangdong Key Laboratory of Mental Health and Cognitive Science, South China Normal University, Guangzhou 510631, China

**Keywords:** graduate students, course support, academic support, thesis writing, anxiety, depression, COVID-19

## Abstract

Because of the impact of the COVID-19 pandemic, the learning style of graduate students has changed considerably, making them more susceptible to psychological problems. This study aimed to explore the mediating roles of thesis writing and anxiety between course support (including course-arrangement, course-assessment, and course-learning), academic support (including academic exchange with colleges, tutors and schoolmates) and depression. There were 3137 graduate students investigated by self-developed Graduate Students’ Academic Affected Questionnaire, Self-Rating Anxiety Scale and Self-Rating Depression Scale. The results showed that (1) 82% of graduate students reported their course support, academic support and thesis writing were affected to varying degrees; (2) course support and academic support correlated with thesis writing, anxiety and depression (*p* < 0.001); (3) the mediation model fitted well, the mediating effect of anxiety between academic support and depression was significant (β = 0.086, SE = 0.02, *p* < 0.001), the serial multiple mediating effects of thesis writing and anxiety between academic support and depression were significant (β = 0.02, SE = 0.008, *p* = 0.013) and the serial multiple mediating effects of thesis writing and anxiety between course support and depression were also found to be significant (β = 0.014, SE = 0.006, *p* = 0.014).

## 1. Introduction

In 2019, COVID-19 broke out and was declared a public health emergency of international concern [1]. The outbreak was large, contagious and deadly, putting great pressure on authorities and individuals. To control the spread of this pandemic, Chinese authorities ordered a strict nationwide lockdown, and the Ministry of Education proposed an initiative, called “Suspending Classes Without Stopping Learning” [2]. Being affected by the pandemic and objective policies, the course pressure on students was increased. In the first half of 2020, schools in 172 countries were forced to close, which affected the education of approximately 1.5 billion students [3]. In China, there were about 200 million elementary and secondary school students who began take online courses in mid-February 2020 [4]. About 30% of students took the classes on time and about 40% of students paid full attention to the lessons after course-arrangement, course-assessment, and course-learning, transforming from in-person teaching to online teaching [5].

Although the learning process was not stopped, COVID-19 had an impact on students’ course-arrangement, course-assessment, course-learning, and academic exchange with colleges, tutors, and schoolmates, which was undoubtedly a challenge for students who were used to in-person classroom learning [6]. Existing research indicated that the sudden onset of coronavirus and transformation from offline teaching to online teaching caused disruptions to students’ academic performance and mental health [7]: preteens and teenagers in the regions severely hit by the pandemic or with family members who were sick were easier to develop anxiety or depression, and some severe cases even felt excessively panicked, uneasy and confused [8]. As for college students, the incidence of negative emotions such as depression and anxiety were reported to be higher than before the outbreak [1,9,10,11,12]. However, existing research mainly investigated elementary, middle-school or college students [13,14,15]. Few studies have explored the impact of the pandemic on graduate students. Jenkins et al. found that individuals with a higher education background were more likely to suffer mental health problems [16]. When the pandemic brought a series of challenges to their courses, graduate students with higher education were more likely to be subjected to mental health problems [16].

Based on the data of Statistical Bulletin on National Education Development in 2020, the total number of students in higher education in China was 41.83 million, including 0.47 million PhDs and 2.67 million masters [17]. There are three specific requirements for graduate students in China, as follows: (1) to obtain the credits of coursework; (2) independently complete a 20,000–50,000-word academic dissertation with independent opinions and the correct application of research methods in related fields; (3) only the publishing of academic papers in journals can be qualified for the graduation project oral defense. Evidently, it is necessary to obtain enough credits from coursework before graduation. Otherwise, the student is considered unqualified and is unable to complete the graduation project oral defense. Furthermore, COVID-19 not only made a difference on graduate students’ course-arrangement, course-assessment and course-learning, moving from offline to online, but also affected the academic exchange with colleges, tutors, and classmates negatively, which may increase the difficulty of graduation for graduate students [18,19,20,21]. Therefore, graduate students with special learning characteristics were more likely to have mental health problems such as anxiety and depression.

### 1.1. Course Support, Academic Support and Depression

Social support typically refers to the functions performed for the individual by significant (i.e., primary) others, divided into subjectively perceived social support and objective social support provided by external sources [22]. Course support and academic support are the crucial elements of social support for graduate students. Taking courses is a key part in the education system. The course support system includes support from course objectives, teaching content, course assessment, teaching resources, and teacher growth [23]. Zhang proposed that course support consisting of course decision support (course-design, course-arrangement, course-assessment, etc.), course resource support (course-knowledge, human and material resources, etc.), course technical support and course-management support was one of the criteria for evaluating the quality of college course activities [24]. Therefore, course support is a sum of various supporting content that benefits students’ learning. In our study, course support was measured by course-arrangement, course-assessment and course-learning.

Academic support offers guidance and support for students in the process of research, such as academic exchange. Chen proposed that academic support includes various guidance activities that play important roles in students’ academic work [25]. Zhang regarded academic support as an academic aid effort provided by teachers, peer students or experts to help students with academic research [26]. In conclusion, academic support means obtaining academic guidance and help from outside. The online study, isolation and other measures during the pandemic have had severe and negative influences on academic exchanges among graduate students, tutors, classmates, and other colleges [20].

Social support is a protective factor for individual mental health and can protect individuals from harmful surroundings [27]. When people receive more social support, they can feel more understood and respected and can maintain their emotions more stably [28] and suffer less anxiety and depression [29]. The susceptibility hypothesis posits that stressful events make individuals prone to depression [30,31,32,33]. Considering course support and academic support are the crucial elements of the social support for graduate students, we suppose that when course support and academic support are negatively affected by the pandemic, depression and other negative emotions may arise in individuals.

### 1.2. The Mediating Effects of Thesis Writing and Anxiety

Finishing an academic dissertation and receiving approval from the degree committee are the requirements for receiving a Master’s degree or a Doctorate. In addition, many universities in China clearly stipulate in their “Graduate Degree Application Regulations” that graduate students have to publish one or more academic papers before graduation [34]. Therefore, graduate students’ thesis writing is highly significant. It is not only the premise for completing a dissertation and obtaining a master’s degree, but also an important means for assisting in academic development [35,36]. Thesis writing is closely related to the course support and academic support. Wei found that scaffolding teaching strategies in academic writing classes could improve students’ thesis writing ability [37]. The existing research about the academic performance of schools and universities indicated that students’ experience of teacher support not only had a positive effect on their learning engagement [38], but could also positively predict their academic achievement [39]. Academic support from tutors can promote graduate students’ thesis writing [40]. Hence, the course support and academic support of graduate students are negatively affected by the pandemic, which would affect students’ thesis writing.

In addition, Du’s research found that some students tended to be anxious, confused, and rabid in the process of thesis writing when there was an unrealistic expectation between targets and actual abilities [41]. For example, some students in English majors have cognitive anxiety in writing essays [42]. Some scholars found that the PhD candidates felt more pressure and anxiety when writing a thesis, which resulted from the confusing topic selection, terrible time management, lack of resources, low writing ability, and the complex interactions between tutors and peers, which may lead to exhaustion and depression [40]. Based on previous studies, we predict that graduate students may feel anxiety in thesis writing due to the pandemic.

Anxiety has a negative impact on individual mental health, and can lead to depression through a variety of mechanisms [43]. Previous studies showed a significantly positive correlation between anxiety and depression and individuals exposed to anxiety were at a higher risk of depression [44,45,46]. The tripartite model, which was proposed by Clark and Watson, showed that there were overlapping symptoms, such as long-term negative emotion and lack of pleasure, among the patients who have anxiety or depression [47]. Moreover, long-term anxiety may trigger individual depression. For example, anxious individuals are prone to social withdrawal behaviors, which further causes individuals to be rejected by their peers, and eventually brings a sense of loneliness and low self-worth. This cognitive vulnerability is a potential risk and the manifestation of an individual depression [48]. That is, individuals who have or have had anxiety disorders are at greater risk of depression. Therefore, once individual course support and academic support were negatively affected by the pandemic, and individuals were further affected in their thesis writing, causing the individual to experience anxiety and ultimately lead to their depression.

### 1.3. Questions and Hypothesis

**Figure 1:**
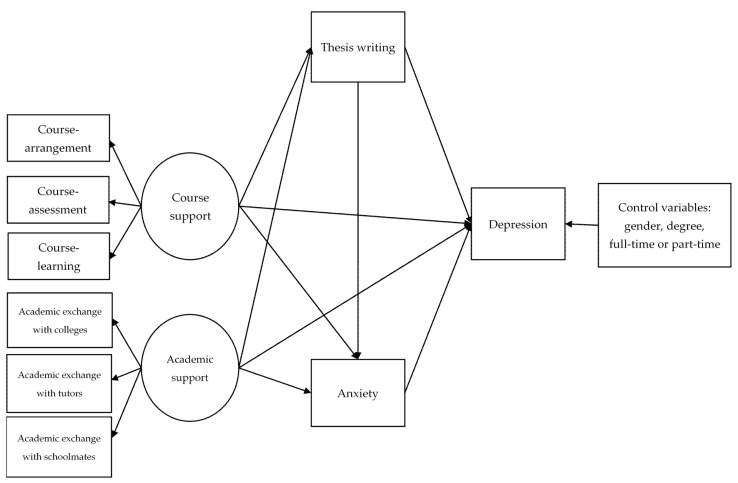
Conceptual model linking Course support, Academic support, Thesis writing, Anxiety and Depression.

In summary, there is a lack of research examining the effect on mental health when social support is negatively affected. The influence of the COVID-19 pandemic on graduate students is even greater, because of their learning characteristics and demanding research work [49]. Hence, exploring the mechanisms of course support, academic support and mental health, which have been affected by the pandemic, can help to develop a theory on the relevant interventions for graduate students. Therefore, based on the social support theory and the tripartite model, our study explored the serial multiple mediating effects of thesis writing and anxiety between course support, academic support and depression. The conceptual framework of this research is shown in Figure 1 and the hypotheses of this research are as follows:

**Hypothesis** **1.** *Negative effects on course support and academic support of graduate students are positively related to anxiety and depression under the COVID-19 pandemic.*

**Hypothesis** **2.** *Thesis writing and anxiety have multiple mediating effects between course support and depression.*

**Hypothesis** **3.** *Thesis writing and anxiety have multiple mediating effects between academic support and depression.*

## 2. Materials and Methods

### 2.1. Participants

This survey was conducted by the team of Professor Zhang from the School of Psychology, South China Normal University. The research concerned graduate students in Chinese universities and the information was collected through an online questionnaire, which consisted of self-developed Graduate Students’ Academic Affected Questionnaire, Self-Rating Anxiety Scale and Self-Rating Depression Scale. According to statistics, a total of 3359 samples were collected from 33 provinces in China. Invalid questionnaires with incorrect answers to the polygraph questions, with an answering time of less than 3 min, and abnormal answers were excluded. There were 3137 valid questionnaires with 93.39% efficiency. The sample was composed of 672 males (21.42%), 2465 female (78.58%); 2829 masters (90.18%), 308 doctors (9.82%); 1442 academic masters (45.97%), 1695 professional masters (54.03%). All survey subjects participated voluntarily by signing an informed consent form. This study was approved by the Ethics Review Committee of South China Normal University, and the ethics application number is SCNU-PSY-2021-021.

### 2.2. Measures

#### 2.2.1. Graduate Students’ Academic Affected Questionnaire by Pandemic

The scale collected the data of graduate students’ exposure to the COVID-19 across three dimensions of course support, academic support and thesis writing. Course support was measured by course-arrangement, course-assessment and course-learning. Cronbach’s α for the scale of course support was 0.89. Academic support was measured via academic exchange with colleges, academic exchange with tutors and academic exchange with schoolmates. Cronbach’s α for the scale of academic support was 0.77. The CFA provided a good fit to the data, RMSEA (Root Mean Square Error of Approximation) = 0.056 (<0.08), SRMR (Standardized Root Mean-Square) = 0.029 (≤0.10), CFI (Comparative Fit Index) = 0.976 (≥0.90) [50]. Cronbach’s α for the total scale of course support and academic support was 0.87.

Thesis writing consisted of one item. The item was “To what extent does the pandemic affect your thesis writing”, which rated on a 4-point scale (1 = no impact, 2 = little impact, 3 = moderate impact, 4 = great impact), with higher scores representing a greater impact.

#### 2.2.2. Self-Rating Anxiety Scale (SAS)

The Self-Rating Anxiety Scale (SAS) was used to assess participants’ anxiety for nearly a week, which was derived from Zung and revised by the Chinese Scale Cooperative Group [51]. The scale has been widely used and showed good reliability and validity in measuring students’ anxiety during the COVID-19 pandemic [33,52,53]. This scale consisted of 20 self-rating items, 15 of which were positive and 5 were negative. Each item was rated on a 4-point scale (1 = never/seldom, 2 = sometimes, 3 = often, 4 = always). Cronbach’s α for the total scale was 0.85.

#### 2.2.3. Self-Rating Depression Scale (SDS)

A Self-Rating Depression Scale (SDS) was used to assess participants’ depression for nearly a week, which was derived from and revised by the Chinese Scale Cooperative Group [54]. The scale showed proper reliability and validity in measuring students’ depression during the COVID-19 pandemic [52,53]. This scale consisted of 20 self-rating items, 10 of which were positive and 10 were negative. Each item was rated on a 4-point scale (1 = never/seldom, 2 = sometimes, 3 = often, 4 = always). Cronbach’s α for the total scale was 0.88.

### 2.3. Statistical Analyses

Following a 3-step procedure for testing the relationship between graduate students’ academic support, academic support, thesis writing and mental health, this study contributed to providing a more comprehensive perspective to explore the psychological mechanism. All variables can be found in Figure 1.

First, descriptive statistics were conducted using SPSS 19.0 (IBM Corp., Armonk, NY, USA) to describe the basic situation of variables, as well as the correlations between variables. Second, a measurement model was constructed, which selected course-arrangement, course-assessment and course-learning as the measurable indicators of course support, selected academic exchange with colleges, tutors and schoolmates as the measurable indicators of academic support. Third, all models were tested under the structural equation modeling framework using Mplus 7.0 (Muthén & Muthén, Los Angeles, CA, USA) [55]. A good fit is indicated by an RMSEA < 0.08, SRMR ≤ 0.10, CFI ≥ 0.90 [50].

## 3. Results

### 3.1. Descriptive Statistics

Table 1 presents the degree of the impact for which each variable is affected by the COVID-19. A total of 82% of graduate students reported their course support, academic support and thesis writing were negatively affected by the COVID-19 to varying degrees. Table 2 presents the means, standard deviations and correlation coefficients among the variables. The results showed that, due to the impact of COVID-19, the indicators of course support (including course-arrangement, course-assessment, course-learning), academic support (including academic exchange with colleges, tutors and schoolmates), thesis writing, anxiety and depression were positively correlated with each other significantly (r = 0.07 to 0.78).

### 3.2. The Measurement Model of Course Support and Academic Support

The measurement model consisted of two latent factors (including course support and academic support), and 6 observed variables (course-arrangement, course-assessment, course-learning, academic exchange with colleges, tutors and schoolmates). The results of the measurement model testing showed a good model fit of χ^2^ (8) = 93.03, *p* < 0.001, RMSEA = 0.058, SRMR = 0.03 and CFI = 0.992. The loadings for the indicators of course support (including course-arrangement, course-assessment, course-learning) were all high (β = 0.83 to 0.87, *p* < 0.001). Additionally, the loadings for the indicators of academic support (including academic exchange with colleges, tutors and schoolmates) were all high (β = 0.51 to 0.88, *p* < 0.001).

### 3.3. The Mediation Model

**Figure 2:**
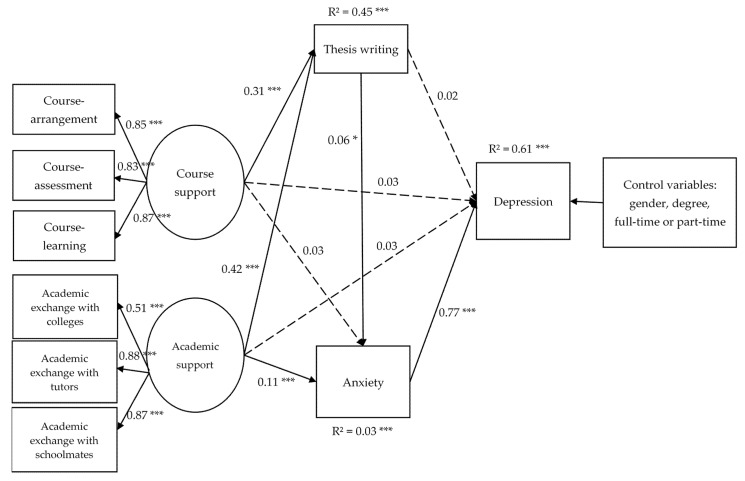
Standardized path coefficients for the mediation model linking Course support and Academic support to post-graduate students depression. Note: Solid lines represent significant paths. Dotted lines represent non-significant path. N = 3137; * *p* < 0.05, *** *p* < 0.001.

This mediation model consisted of gender, degree (academic master’s degree or professional master’s degree) and mode (full-time or part-time) as control variables [56,57,58], course support and academic support as independent variables, thesis writing and anxiety as mediators, and depression as the dependent variable. Figure 2 presents the result of the path analysis. A good fit of χ^2^ (38) = 409.58, *p* < 0.001, RMSEA = 0.056, SRMR = 0.029 and CFI = 0.976 was found for the mediation model.

Due to the impact of COVID-19, (1) the multiple mediating effects of thesis writing and anxiety between course support and depression were significant (β = 0.014, SE = 0.006, *p* = 0.014), indicating that the impact on thesis writing would increase as course support became increasingly affected by the COVID-19 pandemic, triggering individual anxiety further and eventually depression; (2) The direct effect between course support and depression was not significant (β = 0.027, SE = 0.019, *p* = 0.151); (3) The mediating effect of thesis writing between course support and depression was not significant (β = 0.006, SE = 0.005, *p* = 0.212); (4) The mediating effect of anxiety between course support and depression was also not significant (β = 0.026, SE = 0.022, *p* = 0.241).

In addition, due to the negative impact of COVID-19, (1) the mediating effect of anxiety between academic support and depression was significant (β = 0.086, SE = 0.024, *p* < 0.001), indicating that academic support affected by the COVID-19 can trigger individual anxiety further and eventually depression; (2) the multiple mediating effects of thesis writing and anxiety between academic support and depression were significant (β = 0.020, SE = 0.008, *p* = 0.013), indicating that the impact on thesis writing would increase as academic support became further affected by the COVID-19 pandemic, triggering individual anxiety further and eventually depression. (3) The direct effect between academic support and depression was not significant (β = 0.032, SE = 0.020, *p* = 0.105). The mediating effect of thesis writing between academic support and depression was also not significant (β = 0.008, SE = 0.007, *p* = 0.211).

## 4. Discussion

It has been nearly two years since the outbreak of the COVID-19 pandemic began as a public health emergency. Due to the strong contagiousness and the lack of specific medicine for the time being, the pandemic prevention measures have been strict. Although lots of places have been lifted lockdown, the suddenness, urgency, severity of the local pandemic and its high uncertainty, social harmfulness would still trigger anxiety and panic, bringing long-term negative effects on mental health [59]. Previous research on the mental health of school students during COVID-19 have shown that most students have varying degrees of anxiety and depression [1,9,10,11,12]. Wang et al. enrolled 910 graduate students and found that 7.5% of graduate students showed depression and 4.2% of graduate students showed anxiety [59]. The graduate students were in a special stage of development and under heavy pressure of learning and scientific research, which makes them more sensitive to the negative impact of the COVID-19 [49]. In addition, the findings of this study had several implications, which examined the influence of course support and academic support on the mental health of graduate students, based on the social support theory, and further examined the underlying mechanisms by using the tripartite model [47].

### 4.1. The Relation of Course Support, Academic Support and Depression

In this study, graduate students were enrolled as the target participants. The correlation analysis revealed that all indicators of course support and academic support affected by the COVID-19 were positively related to depression. Social support consists of subjective support and objective social support. Subjective social support refers to the perceived respect, support and understanding [22,60], and objective social support refers to the actual support obtained in society (course support and academic support are considered to be objective support in this study). Although previous research has revealed the positive effect of social support on mental health [28,61], less research has examined the effect on mental health when social support is negatively affected. Overall, 82% of graduate students in this study reported the indicators of course support (including course-arrangement, course-assessment, course-learning), academic support (including academic exchange with colleges, tutors and schoolmates) and thesis writing were negatively affected by COVID-19. The protective effect of course support and academic support was reduced, which affected the academic research progress and led to individual mental health problems. Therefore, the course support and academic support of graduate students were important. During the pandemic, colleges should have more reasonable course-arrangement and course-assessment to adapt to the current environment. In addition, tutors and colleges should adopt more active ways to strengthen communication with students and provide more immediate academic support.

### 4.2. The Mediating Effects of Thesis Writing and Anxiety

The results of this study show that, due to the impact of COVID-19, the multiple mediating effects of thesis writing and anxiety between course support and depression were significant, indicating that the impact on thesis writing would increase as course support were negatively affected by the COVID-19 pandemic, further triggering individual anxiety and eventually depression. Affected by COVID-19, students switched from offline learning to online learning, which had a negative impact on course-arrangement, course-assessment and course-learning [5]. Jenkins et al. found that individuals with a higher education background were more likely to suffer from mental health problems [16]. As a group susceptible to mental health problems, graduate students are more likely to feel depression and anxiety during COVID-19, because the pandemic negatively affected course support, resulting in the failure of thesis writing to reach the desired goal on schedule and meet the requirements of academic papers and dissertation for graduation.

The results of this study also found that, due to the impact of COVID-19, the multiple mediating effects of thesis writing and anxiety between academic support and depression were significant, indicating that the impact on thesis writing would increase as academic support became negatively affected by the COVID-19 pandemic, triggering individual anxiety further and depression eventually. The mediating effect of anxiety between academic support and depression was significant, indicating that academic support, as negatively affected by the COVID-19 pandemic, can significantly positively predict individual anxiety and eventually trigger individual depression. It is extremely important for graduate students to cultivate the ability of thesis writing, which is not only a necessary skill to complete the dissertation and obtain the master’s or doctor’s degree, but also is helpful for academic writing and research [35,36]. However, some students were unable to achieve the desired aim on schedule due to the negative effects of less academic support from colleges, tutors and schoolmates during the pandemic, which triggered anxiety and depression.

Anxiety had a negative impact on individual mental health, and those exposed to anxiety had a higher risk of depression [44]. In this study, it was also found that individual who felt anxiety would feel subsequently depression. The tripartite model can be used to explain the result [47], which revealed that anxiety and depression had overlapping symptoms, such as chronic negative emotional states and the lack of pleasure. Anxious individuals may feel belittled, inferior and rejected by their peers. Their cognitive vulnerability may lead to depression over time [48]. Therefore, colleges, social and relevant administrative departments should provide academic support and exchange platforms for graduate students in time during the pandemic. In addition, it is necessary to pay attention to graduate students’ thesis writing, especially the dissertation, so as to avoid the psychological problems caused by the influence of thesis writing. Universities should set up data collection of graduate students’ learning progress, thesis progress and mental health progress during the pandemic period, so as to understand the learning status and psychological dynamics of graduate students in a timely manner, and provide corresponding counseling, guidance and help services.

### 4.3. Implications

Much of the previous research explored social support as a protective factor of individual mental health, but less research examined the effect on mental health when social support was negatively affected. This study explored the way the mental health of graduate students was affected when two types of social support (academic support and social support) were negatively affected by the COVID-19 pandemic. It can help us to understand the impact of the pandemic on graduate students’ mental health from an empirical perspective, and provide theoretical inspirations for psychological intervention under the pandemic. The results showed that, due to the pandemic, the indicators of course support and academic support were negatively affected to varying degrees, which further affected thesis writing, triggering anxiety and eventually depression. It indicated that online communication cannot act as a substitute for offline communication. It also provided practical implications that colleges should not only pay attention in providing course support (such as, setting up courses of thesis writing skills in the form of essay templates and case teaching, arranging course-assessment), but also provide academic online and offline exchange with colleges, tutors and schoolmates, as well as academic reports and self-presentation opportunities. In addition, we should pay attention to the difficulties in data collection and thesis writing, and it is important to offer timely help.

### 4.4. Limitations

However, there are some limitations in this study, which can be improved in future research. Although previous theories and empirical evidence provide a solid foundation, this study was limited in a cross-sectional design, preventing us from drawing causal conclusions. It was also difficult to infer the psychological changes of individuals before and after being affected by the pandemic. Therefore, future research may consider employing lab experimental and longitudinal designs. The second limitation of this study is its reliance on self-reported data from graduate students, which are often biased by social desirability. Therefore, future research needs to consider the multi-method approach.

## 5. Conclusions

This study aimed to explore the serial multiple mediating effects of thesis writing and anxiety between course support, academic support and depression. The results showed that under the pandemic, (1) the more course support and academic support graduate students felt they received, the less their thesis writing was influenced, and the less anxiety and depression they felt.; (2) the impact on thesis writing would increase as course support was affected by the COVID-19 pandemic, triggering individual anxiety further and eventually depression; (3) the impact on thesis writing would increase as academic support became affected by the COVID-19 pandemic, triggering individual anxiety further and eventually depression; (4) depression would increase as academic support was further affected by COVID-19, triggering individual anxiety further. These findings provide theoretical and practical implications for graduate students on the negative impact of the pandemic.

## Figures and Tables

**Table 1:** The impact of COVID-19 on study variables.

Total Sample (N = 3137)	No Impact	Little Impact	Moderate Impact	Great Impact
1. Course-arrangement	14% (n = 438)	30.3% (n = 950)	40.4% (n = 1267)	15.4% (n = 482)
2. Course-assessment	17% (n = 532)	33.8% (n = 1059)	38.6% (n = 1212)	10.6% (n = 334)
3. Course-learning	14.6% (n = 457)	31.3% (n = 981)	39.7% (n = 1246)	14.4% (n = 453)
4. Academic exchange with colleges	8.3% (n = 260)	22.8% (n = 714)	44.9% (n = 1410)	24% (n = 753)
5. Academic exchange with tutors	16.4% (n = 513)	36.6% (n = 1148)	37.2% (n = 1166)	9.9% (n = 310)
6. Academic exchange with schoolmates	17.9% (n = 562)	37.4% (n = 1174)	35.4% (n = 1111)	9.2% (n = 290)
7. Thesis writing	14% (n = 440)	29.7% (n = 932)	39% (n = 1224)	17.2% (n = 541)

Note. N = 3137.

**Table 2:** Means, standard deviations, and correlations among study variables.

Variables	1	2	3	4	5	6	7	8	9	10
1. Gender	-									
2. Course-arrangement	0.007	-								
3. Course-assessment	0.005	0.714 ***	-							
4. Course-learning	0.007	0.734 ***	0.718 ***	-						
5. Academic exchange with colleges	−0.024	0.381 ***	0.356 ***	0.388 ***	-					
6. Academic exchange with tutors	−0.020	0.508 ***	0.490 ***	0.520 ***	0.415 ***	-				
7. Academic exchange with schoolmates	−0.006	0.490 ***	0.473 ***	0.508 ***	0.410 ***	0.771 ***	-			
8. Thesis writing	−0.001	0.500 **	0.461 ***	0.543 ***	0.497 **	0.538 **	0.525 ***	-		
9. Anxiety	0.028	0.119 ***	0.141 ***	0.119 ***	0.069 ***	0.146 ***	0.159 ***	0.152 ***	-	
10. Depression	0.031	0.148 ***	0.156 ***	0.142 ***	0.073 ***	0.160 ***	0.183 **	0.172 ***	0.780 ***	-
M	-	2.57	2.43	2.54	2.85	2.41	2.36	2.59	42.61	47.58
SD	-	0.912	0.893	0.910	0.881	0.875	0.880	0.931	9.853	11.025

Note. N = 3137; ** *p* < 0.01, *** *p* < 0.001.

## Data Availability

The data are not publicly available due to privacy restrictions.

## References

[1] Cao W., Fang Z., Hou G., Han M., Zheng J. (2020). The psychological impact of the COVID-19 epidemic on college students in China. Psychiatry Res..

[2] Office of the Leading Group of the Ministry of Education in Response to the Novel Coronavirus Pneumonia Epidemic Guiding Opinions of the Office of the Leading Group for Responding to the Novel Coronavirus Infection Pneumonia Pandemic of the Ministry of Education on Doing a Good Job in the Organization and Management of Online Teaching in Regular Colleges and Universities during the Period of Pandemic Prevention. http://www.moe.gov.cn/srcsite/A08/s7056/202002/t20200205_418138.html.

[3] UNESCO COVID-19 Impact on Education: From Disruption to Recovery. https://en.unesco.org/covid19/educationresponse.

[4] Ministry of Education Briefs on Online Teaching during COVID-19 and Plan. http://www.gov.cn/xinwen/2020-05/15/content_5511824.html.

[5] Zhang Y., Zhao G.C., Zhou B. (2021). Does learning longer improve student achievement? Evidence from online education of graduating students in a high school during COVID-19 period. China Econ. Rev..

[6] Huang J., Yuan S. (2020). Reflections on Online Learning among College Students during the Outbreak of Novel Coronavirus Pneumonia. J. Chengdu Univ. Tradit. Chin. Med. (Educ. Sci. Ed.).

[7] Dosil Santamaría M., Idoiaga Mondragon N., Berasategi Santxo N., Ozamiz-Etxebarria N. (2021). Teacher stress, anxiety and depression at the 155 beginning of the academic year during the COVID-19 pandemic. Glob. Ment. Health.

[8] Wei H., Chen L., Qian Y., Ke X.Y., Chen Q., Li Y. (2020). Psychological impact of coronavirus disease 2019 on children and adolescents and recommendations for family intervention (1st version). Chin. J. Child Health Care.

[9] Chang H., Yuan Y., Wang D. (2020). Mental health status and its influencing factors among college students during the pandemic of COVID-19. J. South. Med. Univ..

[10] Niu X., Li H., Li J. (2021). The impact of female college students’ pressure perception on anxiety under the back-ground of normalization of COVID-19 prevention and control: A moderated mediation model. China J. Health Psychol..

[11] Odriozola-González P., Planchuelo-Gómez A., Irurtia M.J., de Luis-García R. (2020). Psychological effects of the COVID-19 outbreak and lockdown among students and workers of a Spanish university. Psychiatry Res..

[12] Son C., Hegde S., Smith A., Wang X., Sasangohar F. (2020). Effects of COVID-19 on College Students’ Mental Health in the United States: Interview Survey Study. J. Med. Internet Res..

[13] Chen Y., Cui L., Liu L., Lu F. (2021). Parent-child Conflict among Primary and Middle School Students during the COVID-19 Epidemic and its Countermeasures. Chin. J. Sch. Health.

[14] Currie G., Hewis J., Nelson T., Chandler A., Nabasenja C., Spuur K., Barry K., Frame N., Kilgour A. (2020). COVID-19 Impact on Undergraduate Teaching: Medical Radiation Science Teaching Team Experience. J. Med. Imaging Radiat. Sci..

[15] Moore K.A., Lucas J.J. (2020). COVID-19 Distress and Worries: The Role of Attitudes, Social Support, and Positive Coping during Social Isolation. Psychol. Psychother.: Theory Res. Pract..

[16] Jenkins P., Ducker I., Gooding R., James M., Rutter-Eley E. (2020). Anxiety and depression in a sample of UK college students: A study of prevalence, comorbidity, and quality of life. J. Am. Coll. Health.

[17] National Education Development Statistical Bulletin in 2020. http://www.moe.gov.cn/jyb_sjzl/sjzl_fztjgb/202108/t20210827_555004.html.

[18] Wang Y., Cui X.A. (2020). Research on Satisfaction of Network Teaching of Ideological and Political Course in Higher Education in Context of COVID-19- Undergraduates of Jinan University as Survey Object. J. Open Univ. Guangdong.

[19] Shi X.D., Li R., Huang J.W., Tang Y.M., Xiao H.L., Niu B. (2021). Reflection on the General Education Curriculum: Biosafety and Practice under the Background of Novel Coronavirus Pneumonia. J. High. Educ..

[20] Du W.J., Chen D.W., Pan B., Cao Y.J. (2021). Discussion on the application of “internet+ academic conference” in continuing medical education during the COVID- 19 pandemic. China Med. Educ. Technol..

[21] Santabárbara J., Ozamiz-Etxebarria N., Idoiaga N., Olaya B., BuenoNovitol J. (2021). Meta-Analysis of Prevalence of Depression in Dental Students during COVID-19 Pandemic. Medicina.

[22] Thoits P. (2011). Mechanisms Linking Social Ties and Support to Physical and Mental Health. J. Health Soc. Behav..

[23] Zheng X.Y., Su H.J., Sun L. (2021). Exploration of the Continuous Improvement Mechanism of University Course Quality Based on Core Elements. China Univ. Teach..

[24] Zhang X.L. (2007). The Reflection and Foreseeing of Curriculum Construction of Primary Education Speciality in Universities from the Angle of Curriculum Support. J. Henan Inst. Educ. (Philos. Soc. Sci. Ed.).

[25] Chen W. (2008). Exploration of learning support service for adult higher education. Sci. Technol. West China.

[26] Zhang Q. (2013). The Research on the Undergraduate Academic Support in the United State.

[27] Cohen S. (2004). Social relationships and health. Am. Psychol..

[28] Fredrickson B.L. (2001). The role of positive emotions in positive psychology: The broaden-and-build theory of positive emotions. Am. Psychol..

[29] Galand B., Hospel V. (2013). Peer victimization and school disaffection: Exploring the moderation effect of social support and the mediation effect of depression. Br. J. Educ. Psychol..

[30] Orth U., Robins R.W., Meier L.L. (2009). Disentangling the effects of low self-esteem and stressful events on depression: Findings from three longitudinal studies. J. Personal. Soc. Psychol..

[31] Wu M.Y., Luo X.L. (2021). Research on Psychological Stress Response and Influencing Factors of Medical Students in Colleges and Universities under the Influence of Epidemic Situation. Way Success.

[32] Zhang X.H., Ye T.T., Yao L.J., Song L.G., Wu Z.D. (2019). A psychological behavioral survey among medical students during the outbreak of coronavirus disease. J. Trop. Med..

[33] Wang J., Ding Y., Jiang X.X. (2021). Anxiety and depression levels and coping strategies of college students in Anhui province under the COVID- 19. China J. Health Psychol..

[34] Zhang J., Zhu X.F., Kuang H.S. (2008). Thinking about Setting up Writing Course of Scientific Paper for Postgraduate in Chinese Universities. High. Educ. Forum.

[35] Xiao C., Hu L.L. (2006). On the cultivation of graduate students’ academic ability. Acad. Degrees Grad. Educ..

[36] Ji Y.C. (2019). Integrating Peer Review and Response into Academic Writing in Blending Learning.

[37] Wei L.Q. (2011). An Application of Scaffolding Teaching Strategy to Teaching of English Academic Thesis Writing. Theory Pract. Educ..

[38] Yang Z., Chen Q., Lu T. (2016). The impact of perceived teacher support on study engagement among elementary students in the urban fringe. Mod. Prim. Second. Educ..

[39] Chen Y., Guo S. (2016). Perceived the impact of teacher support on academic achievement among junior middle school students: A mediated moderation effect. Chin. J. Clin. Psychol..

[40] Leila B., Nasrin S., Alireza Y., Nikoo Y. (2016). Management of Stress and Anxiety Among PhD Students During Thesis Writing: A Qualitative Study. Health Care Manag..

[41] Du C.H. (2002). Analysis of Psychological Problems in Writing Graduation Thesis for Self-taught Candidates and Strategies for Guiding. J. Adult Educ. Coll. Hubei Univ..

[42] Li B.Y. (2018). A Study of Thesis Writing Anxiety of English Major Postgraduates.

[43] Garber J., Weersing V.R. (2010). Comorbidity of anxiety and depression in youth: Implications for treatment and prevention. Clinical Psychology: Sci. Pract..

[44] Bittner A., Goodwin R.D., Wittchen H., Beesdo K., Hapfler M., Leib R. (2004). What characteristics of primary anxiety disorders predict subsequent major depressive disorder?. J. Clin. Psychiatry.

[45] Brady E.U., Kendall P.C. (1992). Comorbidity of anxiety and depression in children and adolescents. Psychol. Bull..

[46] Chorpita B.F., Daleiden E.L. (2002). Tripartite dimensions of emotion in a child clinical sample: Measurement strategies and implications for clinical utility. J. Consult. Clin. Psychol..

[47] Clark L.A., Watson D. (1991). Tripartite model of anxiety and depression: Psychometric evidence and taxonomic implications. J. Abnorm. Psychol..

[48] Abramson L.Y., Metalsky G.I., Alloy L.B. (1989). Hopelessness depression: A theory-based subtype of depression. Psychol. Rev..

[49] Zhu H., Si W. (2013). Analysis of current situation, influencing factors and countermeasures of graduate students’ mental health. Party Build. Ideol. Educ. Sch..

[50] Browne M.W., Cudeck R. (1992). Alternative ways of assessing model fit. Sociol. Methods Res..

[51] Zung W.W. (1971). A Rating Instrument for Anxiety Disorders. Psychosomatics.

[52] Wu J.J., Rong X., Chen F., Diao Y.J., Chen D.C., Jing X.C., Gong X.L. (2020). Investigation on sleep quality of first-line nurses in fighting against corona virus disease 2019 and its influencing factors. Chin. Nurs. Res..

[53] Liu Y., Li X.N., Wang X.S., Yang N., Wan B.J., Shi B. (2020). Mediating Effect of Exercise Intervention on Self-Efficacy of Negative Emotion Regulation of Home Schooled Students During the COVID-19. Pandemic. J. Beijing Sport Univ..

[54] Zung W.W.K. (1965). A self-rating depression scale. Arch. Gen. Psychiatry.

[55] Muthén L.K., Muthén B. (2017). Mplus User’s Guide: Statistical Analysis with Latent Variables, User’s Guide.

[56] Li X.G., Wu X.G. (2021). Different Depression in One City: Gender, Class and Mental Health Disparities during COVID-19 in Wuhan. Popul. Dev..

[57] Liu Y.H., Zhang Y.H., Pan X.F. (2021). A Cross-Sectional Meta- Analysis of the Changes in Mental Health of Chinese Postgraduates. J. Neijiang Norm. Univ..

[58] Zhao D.J., Xu J.Y., Sun Q.Z., Zhang Y., Sha J.B. (2004). A Correlative Study on Mental Health of Master Students and Exercise Doing. J. Beijing Sport Univ..

[59] Wang H., Huang Q., Yin H., Guo J., Li Z. (2020). Investigation of Mental Health of Postgraduates and Analysis on Influence Factors during the COVID-19. China J. Health Psychol..

[60] Xiao S. (1994). Theory basis and application on Social Support Rating Scale. J. Clin. Psychiatry.

[61] Yao R., Guo M., Ye H. (2018). The mediating effects of hope and loneliness on the relationship between social support and social well-being in the elderly. Acta Psychol. Sin..

